# CVD-Grown 2D Nonlayered NiSe as a Broadband Photodetector

**DOI:** 10.3390/mi12091066

**Published:** 2021-09-01

**Authors:** Fang Liang, Liangliang Zhan, Tianyu Guo, Xing Wu, Junhao Chu

**Affiliations:** 1 Shanghai Key Laboratory of Multidimensional Information Processing, School of Communication and Electronic Engineering, East China Normal University, Shanghai 200241, China; 52161213023@stu.ecnu.edu.cn (F.L.); 10192100477@stu.ecnu.edu.cn (L.Z.); 10182100136@stu.ecnu.edu.cn (T.G.); 2 State Key Laboratory of Infrared Physics, Shanghai Institute of Technical Physics, Chinese Academy of Sciences, 500 Yutian Road, Shanghai 200083, China

**Keywords:** NiSe, nonlayered materials, photodetector, CVD, 2D materials

## Abstract

Two-dimensional (2D) materials have expansive application prospects in electronics and optoelectronics devices due to their unique physical and chemical properties. 2D layered materials are easy to prepare due to the layered crystal structure and the interlayer van der Waals combination. However, the 2D nonlayered materials are difficult to prepare due to the nonlayered crystal structure and the combination of interlayer isotropic chemical bonds, resulting in limited research on 2D nonlayered materials with broad characteristics. Here, a 2D nonlayered NiSe material has been synthesized by a chemical vapor deposition method. The atomic force microscopy study shows that the grown NiSe with a thin thickness. Energy-dispersive X-ray spectroscopy, X-ray photoelectron spectroscopy and transmission electron microscopy results demonstrate the uniformity and high quality of NiSe flakes. The NiSe based photodetector realizes the laser response to 830 nm and 10.6 μm and the maximum responsivity is ~6.96 A/W at room temperature. This work lays the foundation for the preparation of 2D nonlayered materials and expands the application of 2D nonlayered materials in optoelectronics fields.

## 1. Introduction

Two-dimensional (2D) materials have broad application prospects in the fields of nanoelectronics, optoelectronics, and energy conversion and storage due to their unique physical and chemical properties, such as atomic-scale thickness and ideal bandgap structures [1,2,3,4,5,6,7,8,9,10]. Within them, 2D layered materials have been the most widely studied in recent years owing to the in-plane atoms bonded by strong covalent or ionic bonds and interlayers bonded by weak van der Waals forces, and their smooth surface without chemical dangling bonds, exhibiting excellent electrical, optical and mechanical properties [11,12,13,14]. Ultrathin 2D layered materials are easy to obtain and bandgaps are easy to control benefiting from the unique structure of weak van der Waals forces bonded in interlayers, resulting in great application potential in microelectronics and optoelectronics fields [8,15,16,17,18,19,20,21,22,23,24,25,26,27,28,29,30]. However, 2D non-layered materials with a broad range of properties have rarely been reported in recent years [31,32,33,34]. The essential reason is that it is difficult to obtain ultrathin 2D nonlayered materials with the crystal structure of all atoms bonded by isotropic chemical bonds. Constrained by the surface energy, a layer with an unsaturated dangling bonds surface appears when the thicknesses of the nonlayered crystals are reduced, resulting in the nonlayered materials growing in islands. The preparation of thin nonlayered materials is a key problem that needs to be solved to broaden the applications in the field of nanoelectronics and optoelectronics. The chemical vapor deposition (CVD) method is an effective method for preparing 2D non-layered materials, which can produce thinner materials by adjusting the growth parameters.

In this work, a nonlayered NiSe with a thin flake is successfully synthesized by using a CVD method. The nonlayered NiSe has the hexagonal phase with a hexagonal unit cell belong to the P6_3_/mmc space group with a 6-fold lattice symmetry. The lateral size of the sample is up to 153.8 μm observed by optical microscopy. The energy-dispersive X-ray spectroscopy (EDX) mapping and scanning transmission electron microscopy (STEM) are employed to characterize quality and crystal structure. The composition of grown NiSe flakes is confirmed by EDX and X-ray photoelectron spectroscopy (XPS). The thickness of the product is identified by atomic force microscopy (AFM) technology. The photodetector based on NiSe is fabricated and has laser responses to 830 nm and 10.6 μm. This research enriches the family of infrared photodetectors based on 2D materials and provides the chance to study the interesting properties of 2D nonlayered materials. 

## 2. Materials and Methods

### 2.1. Synthesis of Nonlayered NiSe Flakes

Nonlayered NiSe flakes were synthesized on SiO_2_/Si substrates using the CVD method. The CVD furnace (Thermo Scientific Lindberg/Blue M Moldatherm, Waltham, MA, USA) had a one-inch diameter quartz tube. For the growth of NiSe, nickel dichloride (NiCl_2_) and selenium (Se) powders are employed as reaction precursors. NiCl_2_ powder (99.9%, Alfa Aesar, Ward Hill, MA, USA) is placed in the heating center of the furnace in a quartz boat. The polished surface of the cleaned SiO_2_/Si substrate is placed face down on the top of the quartz boat containing the NiCl_2_ powder. A quartz boat with 0.5 g Se powders (99.99%, Sigma-Aldrich, Burlington, MA, USA) is placed upstream at the edge of the furnace. The distance between the two precursors is about 10.8 cm. Before growing, the CVD furnace is purified with 200 standard cubic centimeters (sccm) per minute of argon (Ar) for one hour. Then the temperature of the furnace is ramped up from room temperature to 680 °C in 22 min in an Ar atmosphere and kept at 680 °C for 15 min for growing NiSe flakes. Ar with a flow rate of 80 sccm is employed as the carrier gas during the growing process. After growth, the furnace is cooled down to room temperature naturally.

### 2.2. Characterization Tools 

The optical images of NiSe flakes are obtained by using an optical microscope (BX41M-LED, OLYMPUS, Tokyo, Japan). Elemental analysis of NiSe materials is performed via XPS technology (AXIS UltraDLD, Kratos, UK). The layer thicknesses of the NiSe flakes are determined by using AFM (MicroNano D-5A). The EDX characterization is carried out on a field emission scanning electron microscope (GeminiSEM 450, ZEISS, Oberkochen, Germany) facility with an acceleration voltage of 5 kV. Raman spectroscope (LabRAM HR-800, Horiba, Kyoto, Japan) with a laser excitation wavelength of 532 nm was employed to characterize the Raman spectra of the samples. The grating was 1800 lines mm-1. The laser beam was focused by a 50× objective on the samples. The transmission electron microscope (TEM) sample is prepared by using an isopropanol-assisted transfer technology. The STEM images were recorded by an FEI Talos F200X system (Thermo Scientific, Waltham, MA, USA) with an acceleration voltage of 300 kV. 

### 2.3. Fabrication of NiSe Device

For the fabrication of NiSe device, firstly, the NiSe flakes on a SiO_2_/Si substrate were spin-coated with polymethyl methacrylate (PMMA; 495 K, A4) at a speed of 4000 rpm with 40 s and then solidified with 5 min under 100 °C on a hot plate. Electron-beam lithography (EBL, FEI F50 SEM equipped with a NPGS system) is employed to draw the electrode patterns. Then, the contact electrodes Cr/Au (20/80 nm) metal films are deposited by electron-beam evaporation. Finally, NiSe device is fabricated after the standard lift-off process. The NiSe devices are wire-bonded onto a supercontinuum light source (sc-pro, YSL Photonics, Wuhan, China) to perform the electrical tests and photoresponse tests. Laser with a wavelength of 830 nm is focused by a 20× objective lens. A commercial CO_2_ laser source (λ = 10.6 μm) is used as a long-wavelength infrared light source. All NiSe devices are measured at room temperature in an ambient environment. 

## 3. Results

### 3.1. The Physical Properties of NiSe Flakes

**Figure 1:**
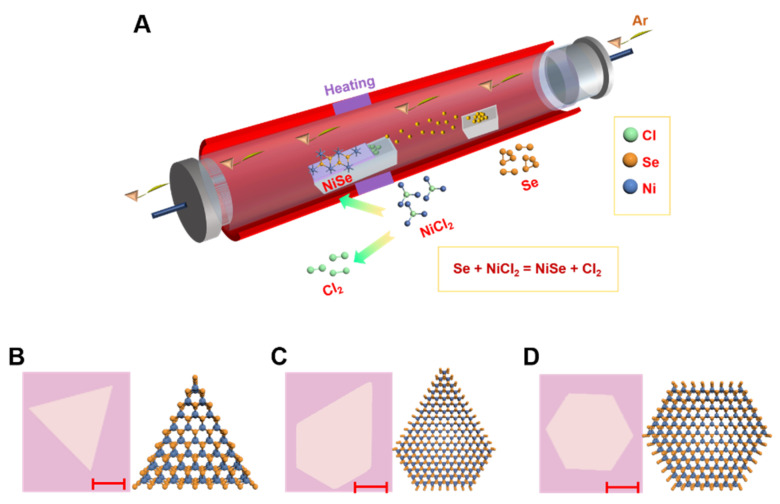
The growth of NiSe flakes with a chemical vapor deposition (CVD) method. (**A**) Schematic of the CVD setup for NiSe synthesis. The precursors are NiCl_2_ and Se powers. (**B**) The optical image and corresponding atomic structure image of triangle-like NiSe flake. (**C**) The optical image and corresponding atomic structure image of pentagon-like NiSe flake. (**D**) The optical image and corresponding atomic structure image of hexagon-like NiSe flake. The scale bar is 10 um.

The NiSe materials were synthesized on SiO_2_/Si substrates by the CVD method. Figure 1A shows the schematic of the CVD setup for growing NiSe flakes. The growth of NiSe in the high temperature mainly goes through four steps:

- The NiCl_2_ powders and Se powders began to sublime as NiCl_2_ vapor and Se vapor and were transported downstream by the carrier gas Ar when the temperature of the furnace arrived at 680 °C;
- The NiCl_2_ vapor and Se vapor diffused toward the substrates;
- The NiCl_2_ vapor and Se vapor adsorbed onto the surface and the adatoms diffused along the surface of the substrate;
- The NiCl_2_ vapor and Se vapor react to form NiSe structures onto the substrate and the other product chlorine gas (Cl_2_) flowed out of the tube along with carrier gas.

The chemical equation between NiCl_2_ and Se is shown in Equation (1):

(1) $${\mathrm{NiCl}}_{2}+\mathrm{Se}\to \mathrm{NiSe}+{\mathrm{Cl}}_{2}$$

Morphology studies are important because the morphology has an important effect on the catalytic performance of NiSe. In our experiment, different morphologies of NiSe flakes were formed. The different morphologies are grown randomly. Figure 1B–D show the optical images and corresponding atomic structure images of triangle-like NiSe, pentagon-like NiSe, and hexagon-like NiSe, respectively. Different factors may influence the morphologies of NiSe flakes produced by a CVD method, such as the growth temperature, the growth time, the distance between the NiCl_2_ and Se powders, the flow rate of carrier gas, and the concentration of precursors. The essential factor for morphologies is the edge diffusion barrier of atoms. The high edge diffusion barrier leads to irregular morphologies [35]. The NiSe with hexagon shape was formed when the growth rate of different edge terminations was equal. The pentagon shape NiSe may be formed as a result of the cyclic twinning mechanism. The optical image in Figure S1a shows the high quality and uniform NiSe flakes. The NiSe sample marked in a yellow rectangle in Figure S1b is with a maximum lateral size of 153.8 μm. The optical image with black spots on the surface of the flakes in Figure S1c may indicate that the grown non-layer NiSe flakes may be not stable in the air.

**Figure 2:**
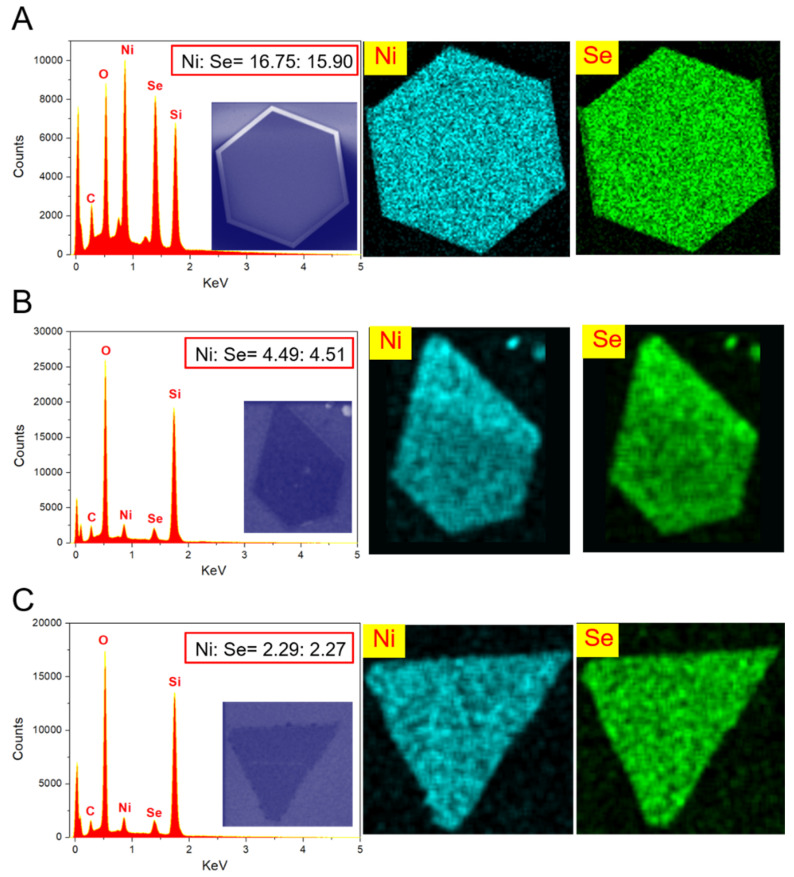
The energy-dispersive X-ray spectroscopy (EDX) characterization and mapping images of as-grown NiSe flakes. (**A**) The EDX curve and corresponding mapping images of hexagon-like NiSe flake. (**B**) The EDX curve and corresponding mapping images of pentagon-like NiSe flake. (**C**) The EDX curve and corresponding mapping images of triangle-like NiSe flake. Insets show the SEM images.

**Figure 3:**
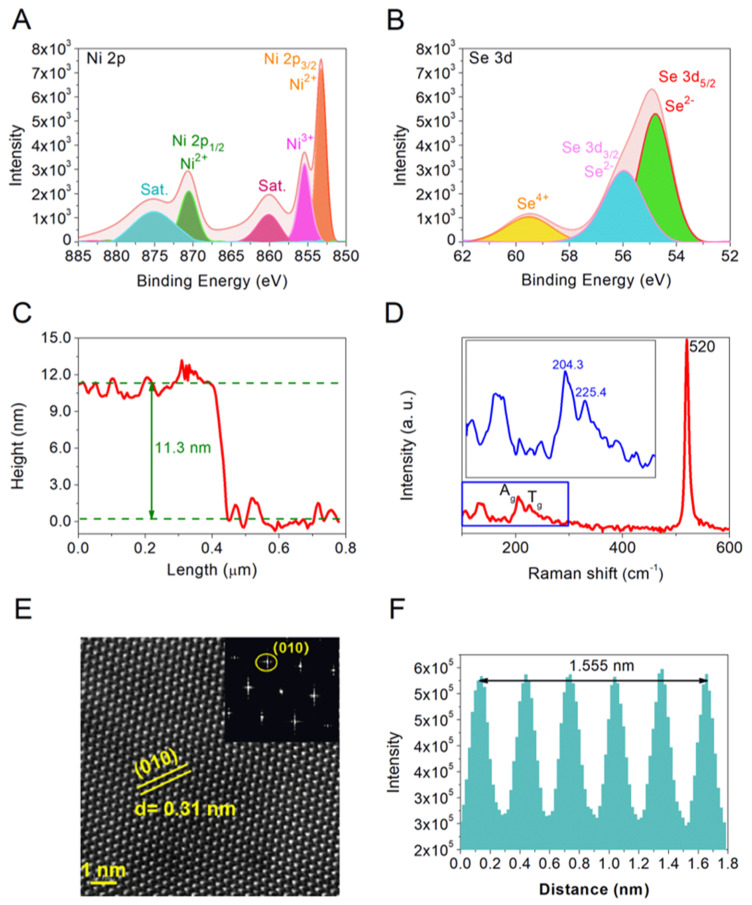
The characterization of as-grown NiSe flakes. (**A**) High-resolution XPS spectrum of grown NiSe in Ni 2p regions. (**B**) High-resolution XPS spectrum of grown NiSe in Se 3d regions. (**C**) Height profile of as-grown NiSe characterized by AFM technology. (**D**) Raman spectra of as-grown NiSe samples with a laser excitation wavelength of 532 nm. (**E**) STEM patterns of NiSe flakes on a copper mesh, the scale bar is 1 nm. The lattice distance along the crystal plane (010) is 0.31 nm. The top right inset of the image shows the corresponding selected area electron diffraction pattern (SAED), which demonstrates the hexagon structure of grown NiSe flakes. (**F**) Lattice distance of (010) plane in Figure 3E.

Common characterization tools are used to further study the properties of as-grown samples. The EDX and SEM technologies are used to characterize the chemical composition and uniformity. As shown in Figure 2, The EDX spectra of as-grown samples with triangle shape (Figure 2A), pentagon shape (Figure 2B), and hexagon shape (Figure 2C) show the atoms ratios of Ni: Se is nearly 1:1, which proves the grown materials are NiSe flakes. The Ni/Se ratio is controlled by the growth temperature. The same atomic ratios of the as-grown materials indicate that the grown materials are the same, which proves the stability of the experimental conditions and the compositional homogeneity. The corresponding mapping images indicate that the grown NiSe flakes are uniformity and NiSe with hexagon shape is of better quality. XPS technology is further used to study the elemental composition and valence states of the grown sample. Figure 3A,B show the XPS spectra of high-resolution Ni 2p and Se 3d, respectively. The two peaks located at 870.5 and 853.2 eV in Figure 3A are is attributed to Ni 2p_1/2_ and Ni 2p_3/2_, respectively, which is corresponding to Ni^2+^. The satellite peaks (Sat.) located at 860.1 and 875.1 eV derived from nickel oxide as the surface exposed to air and oxidized. The peak at 855.4 eV is consistent with Ni^3+^ [36]. The overlapping peaks located at 55.9 and 54.8 eV in Figure 3B are assigned to Se 3d_3/2_ and Se 3d_5/2_, respectively, corresponding to Se^2−^. The XPS results further confirm that NiSe flakes have been successfully synthesized. To study the thicknesses of as-grown NiSe flakes, the representative AFM technology is employed. As shown in Figure 3C, the thickness of the thin flake NiSe in this experiment is 11.3 nm. The corresponding optical image and recognition image are shown in Figure S1d. The recognition image indicates the smooth surface morphologies of the prepared NiSe. The characterized Raman spectra of as-prepared NiSe samples with a laser excitation wavelength of 532 nm are shown in Figure 3D. The prominent peak locating at 520 cm^−1^ is consistent with the Si peak. The Raman peak occurring around 204.4 and 225.4 cm^−1^ correspond to the A_g_ and T_g_ mode, respectively, which are in line with the stretching and librational modes or their combination of Se–Se pairs. The Raman peaks are different from that of NiSe_2_. There are no obvious peaks that occur in 141 and 235 cm^−1^, indicating the amorphous Se element is not synthesized. The absence of the peak around 506 cm^−1^ indicates that there are almost no defects and surface effects on the surface of the prepared NiSe sample [37]. The Raman spectra demonstrate high-quality NiSe samples have been successfully synthesized. TEM technology is further employed to characterize the quality and lattice structures of as-synthesized NiSe flakes. The TEM samples are prepared by transferring the grown NiSe flakes onto a copper grid with an isopropanol-assisted method. As shown in Figure 3E, the STEM image indicates a clear hexagonal symmetry structure and high quality of the prepared NiSe sample. The one set of diffraction spots with 6-fold symmetry shown in the selected area electron diffraction (SAED) image on the top right of STEM pattern demonstrates the single-crystal property of NiSe flakes with high quality and good crystallinity. As shown in Figure 3F, the interplanar distance of the NiSe sample measured in Figure 3E is about 0.31 nm, which is consistent with the 010 planes. All characterization results exhibit the thin NiSe flakes with high quality, good crystallinity, and uniform surface are successfully synthesized.

### 3.2. The Optoelectronic Properties of NiSe Devices

**Figure 4:**
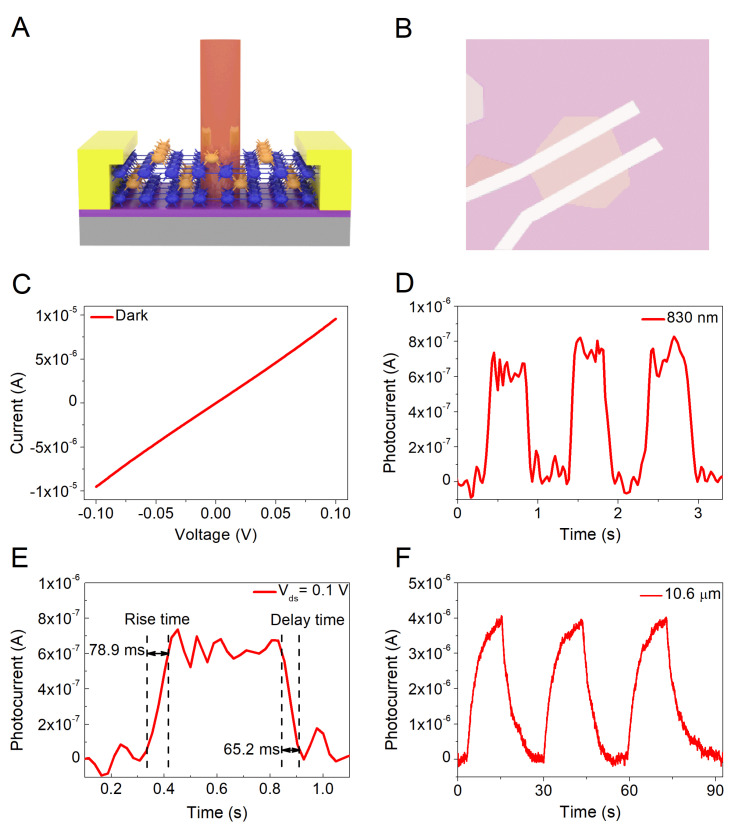
The Photoelectric performance of the NiSe based detector. (**A**) Schematic image of the NiSe photodetector. (**B**) The optical image of the fabricated NiSe device. (**C**) The output curve of a NiSe photodetector without light illumination. (**D**) The time-resolved photoresponse under an 830 nm wavelength illumination at 0.1 V bias. (**E**) The rise time and delay time extracted from the time-resolved photocurrent. (**F**) The time-resolved photoresponse under a 10.6 um wavelength illumination at 0.1 V bias.

To investigate the intrinsic optoelectronic characteristics of the grown NiSe flakes, the NiSe-based detectors were fabricated. The schematic illustration of the NiSe device is shown in Figure 4A. The metal electrodes with 20 nm Cr and 80 nm Au are fabricated by the electron-beam lithography technology followed by the electron-beam evaporation process. The channel length of the NiSe device is about 4 μm. The effective illumination area of the device is about 50.4 μm^2^. Figure 4B shows the corresponding optical image of the NiSe device. The source-drain current-voltage characteristics of the NiSe-based detector in Figure 4C demonstrate good contact between the electrodes and as-grown samples. The optoelectronic properties of the NiSe detector were studied by illuminating with different lasers. Figure 4D,F indicates the time-resolved photoresponse with the light on/off photoswitching behavior of the NiSe detector under illumination lasers of 830 nm, and 10.6 um wavelength at *V_ds_* = 0.1 V, respectively. Here, the generated photocurrent is stable with 830 nm laser illumination and increasing with 10 um laser illumination. The difference may be the following reasons: (1) Lots of photo-generated carriers appear in the channel when the incident laser with 830 nm illuminates on the NiSe sample. The photogenerated electrons and holes that existed in the channel are separated under the external electric field to form the photocurrent. The photocurrent value is stable when the carrier concentration is unchangeable. This is the reason for the photocurrent shows a plateau after 830 nm laser illumination. (2) The increasing trend under 10 um laser illumination may be caused by the photoelectric effect accompanied by the thermal effect. The thermal effect may be due to the high power of the 10um laser source. The thermal effect results in the changeable carrier concentration which causes the increased photocurrent. Figure 4E shows the response time of the NiSe detector which is extracted from Figure 4D. The response time is a physical quantity to describes how fast the NiSe detector responds to laser radiation power. The rise time in Figure 4E is ~78.9 ms and the decay time is ~65.2 ms. Photoresponsivity (*R*) is an important performance indicator of photodetectors, which determines the application fields of the NiSe detector. *R* describes the ability to use the signal generated by the unit radiation power incident on the detector and characterizes the sensitivity of the photodetector to laser illumination. As shown in equation 2, *R* can be calculated by the following expression:

(2) $$R=I_{\mathrm{ph}}/P,$$

where *I*_ph_ is the photocurrent, and *P* is the incident power defined as:

(3) $$P=P_{\mathrm{in}}A,$$

where *P*_in_ is the incident power density, and *A* is the effective illumination area of the device. the *R* for the NiSe detector under 830 nm wavelength illumination is 11 mA/W with an incident power of 61.6 μW. The *R* for the NiSe detector to10.6 μm laser wavelength with 0.57 μW incident power is 6.96 A/W, which is the maximum responsivity of the NiSe device. Specific detectivity (D*) is an important indicator of the detector’s ability to detect the minimum signal, it can be calculated as:

(4) D*=R4TKBAR0+2qIdarkA,

where *R* is the photoresponsivity, *T* stands for the temperature, *K_B_* represents the Boltzmann constant, A is the effective illumination area, *R_0_* acts as the resistance, *q* is the elementary electronic charge, and *I_dark_* is the dark current of the NiSe device. The D* of the NiSe detector under 10.6 μm wavelength illumination is 2.3 × 10^7^ cm·Hz^1/2^/W. The fabricated NiSe photodetector realizes the laser response to 830 nm and 10.6 μm for the first time. 

Table S1 summarizes the *R* and D* of 2D nonlayered materials under different lasers illumination. Although the specific detectivity is not ideal, the photoresponsivity of NiSe based photodetector is considerable and we have realized the photoresponse of nonlayered NiSe flakes to mid-infrared wavelength 10.6 um.

## 4. Discussion and Conclusions

In summary, 2D nonlayered NiSe flakes have been synthesized successfully by using a CVD method in this work. The thickness of the as-grown NiSe flake identified by AFM technology is about 11.3 nm. The maximum lateral size of the NiSe flake is up to 153.8 μm. The STEM and EDX results indicate the high quality of the grown NiSe. The fabricated NiSe-based photodetector exhibits considerable photoresponse to 830 nm and 10.6 μm laser wavelength. The NiSe-based photodetector shows a considerable photoresponse speed, with a rise time of 78.9 ms and decay time of 65.2 ms. The photoresponsivity of the NiSe-based photodetector under 10.6 μm laser illumination is ~ 6.96 A/W and the corresponding specific detectivity is 2.3 × 10^7^ cm·Hz^1/2^/W. This work provides a potential candidate material for infrared photoelectronic devices. It is necessary to further study the influence of thicknesses and crystal structures of 2D nonlayered NiSe on the performance of the device and to further improve the optoelectronic performance of the NiSe-based photodetector.

## Supplementary Materials

The following are available online at https://www.mdpi.com/article/10.3390/mi12091066/s1, Figure S1: The optical images and AFM results of grown NiSe flakes, Table S1: The summaries of nonlayered materials-based photodetectors.
