# Supplementary material for: CVD-Grown 2D Nonlayered NiSe as a Broadband Photodetector
Source: Micromachines (Basel). 2021 Sep 1;12(9):1066. doi: 10.3390/mi12091066 (PMC8471629; doi:10.3390/mi12091066)
Supplement: Supplementary file 1 [file micromachines-12-01066-s001.zip › micromachines-1364658-supplementary.pdf]

Supplementary Materials: CVD-Grown 2D Nonlayered NiSe as a Broadband Photodetector

Fang Liang [1], Liangliang Zhan [1], Tianyu Guo [1], Xing Wu [1,*] and Junhao Chu [1,2,*]

1 Shanghai Key Laboratory of Multidimensional Information Processing, School of Communication and Electronic Engineering, East China Normal University, Shanghai, 200241, China; 52161213023@stu.ecnu.edu.cn (F.L.); 10192100477@stu.ecnu.edu.cn (L.Z.); 10182100136@stu.ecnu.edu.cn (T.G.)

2 State Key Laboratory of Infrared Physics, Shanghai Institute of Technical Physics, Chinese Academy of Sciences, 500 Yutian Road, Shanghai, 200083, China

* Correspondence: xwu@cee.ecnu.edu.cn (X.W.); jhchu@mail.sitp.ac.cn (J.C.); Tel.: +86-189-1818-0597 (X.W.)

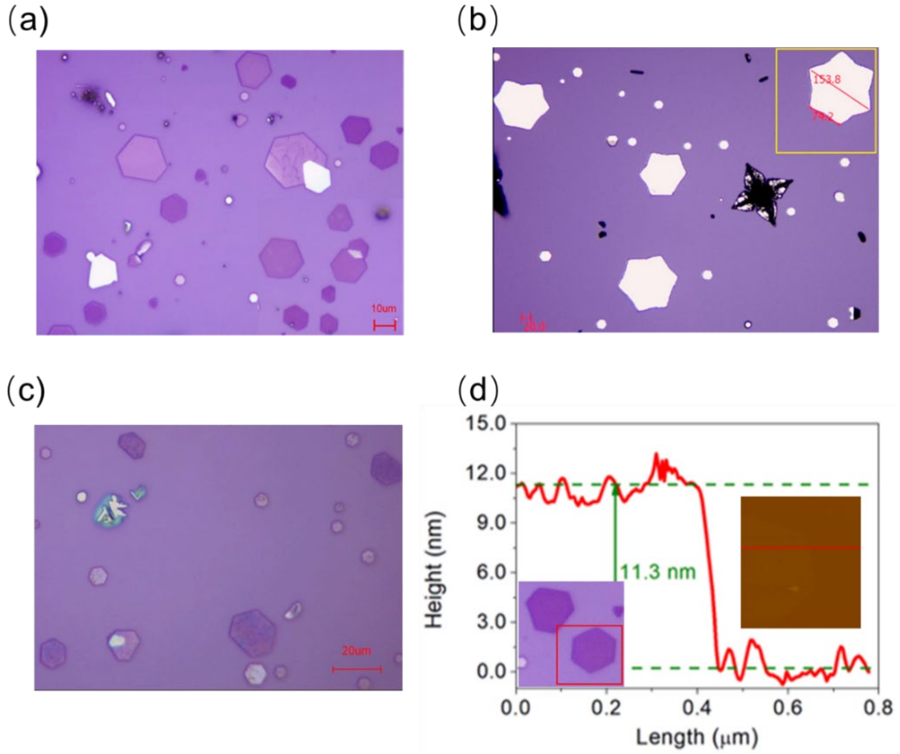

Figure S1 The optical images and AFM results of grown NiSe flakes. (**a**) The optical images of NiSe flakes with different morphologies. (**b**) The optical image of NiSe flakes with 153.8 um. (**c**) The optical image of NiSe flakes grown one month ago. (**d**) The optical image of thinner NiSe flake and the corresponding AFM result with height profile and recognition image.

Table S1. The summaries of nonlayered materials-based photodetectors.

Materials	Laser wavelength (nm)	Voltage (V)	*R* (A/W)	*D** (Jones)	Ref.
PdS	400	2	0.05	7.81×10^8	[1]
CdTe	473	1	0.6×10^{-3}	-	[2]
β-In_2S_3	450	1	137	4.74×10^{10}	[3]
Te	350	0.6	1.34×10^{-5}	3.1×10^7	[4]
$Pb_{1-x}Sn_xSe$	473	2	5.95	-	[5]
γ-Ga_2S_3	350	1	61.3	1.52×10^{10}	[6]
ZnSb	1550	0.5	89.2	1.7×10^{10}	[7]
CoSe	10.6×10^3	1	2.58	3.3×10^9	[8]
NiSe	10.6×10^3	0.1	6.96	2.3×10^7	This work

References

1. Jin, B.; Huang, P.; Zhang, Q.; Zhou, X.; Zhang, X.; Li, L.; Su, J.; Li, H.; Zhai, T., Self-Limited Epitaxial Growth of Ultrathin Nonlayered CdS Flakes for High-Performance Photodetectors. *Advanced Functional Materials* **2018,** 28, (20), 1800181.
2. Cheng, R.; Wen, Y.; Yin, L.; Wang, F.; Wang, F.; Liu, K.; Shifa, T. A.; Li, J.; Jiang, C.; Wang, Z.; He, J., Ultrathin Single-Crystalline CdTe Nanosheets Realized via Van der Waals Epitaxy. *Adv Mater* **2017,** 29, (35).
3. Huang, W.; Gan, L.; Yang, H.; Zhou, N.; Wang, R.; Wu, W.; Li, H.; Ma, Y.; Zeng, H.; Zhai, T., Controlled Synthesis of Ultrathin 2D β-In_2S_3 with Broadband Photoresponse by Chemical Vapor Deposition. *Advanced Functional Materials* **2017,** 27, (36), 1702448.
4. Xie, Z.; Xing, C.; Huang, W.; Fan, T.; Li, Z.; Zhao, J.; Xiang, Y.; Guo, Z.; Li, J.; Yang, Z.; Dong, B.; Qu, J.; Fan, D.; Zhang, H., Ultrathin 2D Nonlayered Tellurium Nanosheets: Facile Liquid-Phase Exfoliation, Characterization, and Photoresponse with High Performance and Enhanced Stability. *Advanced Functional Materials* **2018,** 28, (16), 1705833.
5. Wang, Q.; Xu, K.; Wang, Z.; Wang, F.; Huang, Y.; Safdar, M.; Zhan, X.; Wang, F.; Cheng, Z.; He, J., van der Waals epitaxial ultrathin two-dimensional nonlayered semiconductor for highly efficient flexible optoelectronic devices. *Nano Lett* **2015,** 15, (2), 1183-9.
6. Zhou, N.; Gan, L.; Yang, R.; Wang, F.; Li, L.; Chen, Y.; Li, D.; Zhai, T., Nonlayered Two-Dimensional Defective Semiconductor gamma-Ga_2S_3 toward Broadband Photodetection. *ACS Nano* **2019,** 13, (6), 6297-6307.
7. Chai, R.; Chen, Y.; Zhong, M.; Yang, H.; Yan, F.; Peng, M.; Sun, Y.; Wang, K.; Wei, Z.; Hu, W.; Liu, Q.; Lou, Z.; Shen, G., Non-layered ZnSb nanoplates for room temperature infrared polarized photodetectors. *Journal of Materials Chemistry C* **2020,** 8, (19), 6388-6395.
8. Liang, F.; Wang, C.; Luo, C.; Xia, Y.; Wang, Y.; Xu, M.; Wang, H.; Wang, T.; Zhu, Y.; Wu, P.; Ye, J.; Mu, G.; Zhu, H.; Wu, X., Ferromagnetic CoSe broadband photodetector at room temperature. *Nanotechnology* **2020,** 31, (37), 374002.
